# Surface Modification of LiNi_0.8_Co_0.15_Al_0.05_O_2_ Particles via Li_3_PO_4_ Coating to Enable Aqueous Electrode Processing

**DOI:** 10.1002/cssc.202001907

**Published:** 2020-10-07

**Authors:** Michael Hofmann, Felix Nagler, Martina Kapuschinski, Uwe Guntow, Guinevere A. Giffin

**Affiliations:** ^1^ Fraunhofer Institute for Silicate Research ISC Fraunhofer R&D Center Electromobility Neunerplatz 2 97082 Würzburg Germany

**Keywords:** aqueous electrode processing, lithium-ion battery, lithium phosphate coating, Ni-rich cathode material, sustainable chemistry

## Abstract

The successful implementation of an aqueous‐based electrode manufacturing process for nickel‐rich cathode active materials is challenging due to their high water sensitivity. In this work, the surface of LiNi_0.8_Co_0.15_Al_0.05_O_2_ (NCA) was modified with a lithium phosphate coating to investigate its ability to protect the active material during electrode production. The results illustrate that the coating amount is crucial and a compromise has to be made between protection during electrode processing and sufficient electronic conductivity through the particle surface. Cells with water‐based electrodes containing NCA with an optimized amount of lithium phosphate had a slightly lower specific discharge capacity than cells with conventional *N*‐methyl‐2‐pyrrolidone‐based electrodes. Nonetheless, the cells with optimized water‐based electrodes could compete in terms of cycle life.

## Graphical abstract

**The coating amount is the decisive factor**: Environmentally friendly aqueous electrode processing of highly water‐sensitive LiNi_0.8_Co_0.15_Al_0.05_O_2_ (NCA) particles is possible with a protective lithium phosphate surface coating. By using an optimized coating amount, excellent electrochemical performance of cells with water‐based electrodes can be achieved, which can compete with that of cells with conventional state‐of‐the‐art electrodes.

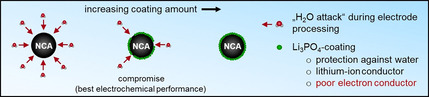

## Introduction

Lower overall battery costs and environmental impact of current and next‐generation lithium‐ion battery production are two critical aspects in the development of lithium‐ion batteries. The implementation of a water‐based manufacturing process seems to be a promising approach to address these points. It can lower the electrode production costs and therefore the overall battery price. At the same time, it would improve the environmental benignity of battery production.^[1]^ The use of an aqueous process would enable the elimination of the mutagenic and toxic solvent *N*‐methyl‐2‐pyrrolidone (NMP), which, as of yet, remains an integral component of state‐of‐the‐art cathode electrode processing. From an environmental perspective, the regulations concerning the use of NMP in the US^[2]^ and Europe^[3]^ have been made more stringent. From a cost perspective, a water‐based process should allow savings in terms of the solvent/binder prices, along with reduced investment and operation costs for the production plant.[^1^, ^4^, ^5^]

Despite the advantages, aqueous processing for the cathode materials is still problematic as metal leaching from the cathode active material and the resultant highly alkaline slurries lead to the production of surface impurities, a delithiated subsurface, and aluminum current collector corrosion, which have a detrimental effect on the electrochemical performance.[^1^, ^6^, ^7^, ^8^, ^9^, ^10^, ^11^, ^12^, ^13^, ^14^, ^15^] Various strategies with diverse cathode materials have been developed to improve aqueous‐manufactured electrodes, wherein the modification of the active material by applying surface coatings seems to be very promising.[^6^, ^7^, ^8^, ^9^, ^10^, ^11^, ^16^]

LiNiCoAlO_2_ (NCA) has attracted significant attention as a cathode active material because of its high energy density.^[17]^ However, NCA is known to be extremely sensitive to moisture,[^15^, ^18^, ^19^, ^20^] making it a difficult candidate for the aqueous electrode processing. According to the results in the literature, it is assumed that aqueous processing of NCA will not be successful without additional surface modifications, prior to electrode fabrication[^10^, ^11^] or in situ surface modification during processing[^8^, ^9^] or in a combination of both.^[21]^

The strong P−O‐bonding energy in the PO_4_^3−^ ion gives metal phosphates high structural stability against chemical attack.[^22^, ^23^, ^24^] Various phosphate coatings such as Ni_3_(PO_4_)_2_,^[25]^ FePO_4_,^[26]^ LiMnPO_4_,^[27]^ MgHPO_4_,^[28]^ BiPO_4_,^[29]^ Li_1.3_Al_0.3_Ti_1.7_(PO_4_)_3_,^[30]^ Li_3_PO_4_,[^23^, ^31^] Co_3_(PO_4_)_2_,[^32^, ^33^] LiFePO_4_,^[34]^ and AlPO_4_[^31^, ^33^, ^35^] have been studied on NCA and resulted in improved electrochemical performance. However, to the best of the authors’ knowledge, a phosphate‐coated NCA has never been used in a combination with a water‐based electrode manufacturing process. Amongst the phosphate coatings mentioned above, Li_3_PO_4_ is relatively easy to synthesize and, in contrast to other metal phosphates such as Ni_3_(PO_4_)_2_, Co_3_(PO_4_)_2_, BiPO_4_, FePO_4_, MgHPO_4_, and AlPO_4,_ a lithium‐ion conductor.[^36^, ^37^] The latter aspect might comparatively facilitate the migration of lithium ions through the particles surface.

Therefore, in this study, the surface of LiNi_0.8_Co_0.15_Al_0.05_O_2_ particles was modified by applying Li_3_PO_4_ coatings via a simple precipitation reaction. The modified particles are compared with pristine NCA in terms of their processability in water and their electrochemical performance in cells. Finally, the cycle stability of cells with electrodes prepared via an aqueous and the conventional NMP route as reference is investigated.

## Results and Discussion

The lithium phosphate coating of NCA was carried out via a simple precipitation reaction. The chemical reaction is assumed to be as follows [Eq. (1)]:

(1) $$3\;\mathrm{CH}{}_{3}\mathrm{COOLi}+H{}_{3}\mathrm{PO}{}_{4}\to \mathrm{Li}{}_{3}\mathrm{PO}{}_{4}\;↓+3\;\mathrm{CH}{}_{3}\mathrm{COOH}$$

The coating process was done using ethanol as a solvent to reduce contact of the active material with water in order to minimize pre‐damage of the particle during the coating process.

X‐ray diffraction (XRD) of the pristine and lithium phosphate‐coated powders was performed to determine if the coating had an effect on the NCA bulk crystal structure (Figure S1). All peaks of the pristine NCA can be assigned to a layered α‐NaFeO_2_ structure with *R*3 *m* space group with clear peak separations of the 006/012 and 108/110 reflections, indicating a highly ordered structure.^[38]^ For the coated powder, no change in the XRD pattern and no impurity phase peaks were detected. Thus, the coating process does not affect the active material bulk structure. Peaks attributable to the diffraction pattern of Li_3_PO_4_ are not found in the XRD spectra of the coated samples, likely due to the low coating content.

**Figure 1:**
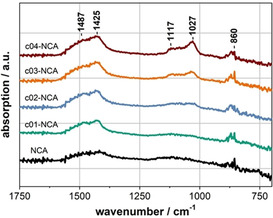
ATR‐FTIR spectra of pristine and coated NCA particles.

To confirm the presence of lithium phosphate, the pristine and coated‐NCA particles were characterized by attenuated total reflection Fourier‐transform infrared (ATR‐FTIR) spectroscopy. The magnified spectra (Figure 1) of the pristine and coated NCA particles show peaks around 860, 1425, and 1487 cm^−1^, which can be assigned to the CO‐bending, symmetric CO‐stretching, and asymmetric CO‐stretching vibrations, respectively.^[19]^ These peaks can be attributed to carbonate species, which are often detected on the surface of layered Ni‐rich oxides and can be formed by various processes.[^39^, ^40^, ^41^] For the coated samples, two additional peaks evolve with increasing coating content at around 1027 and 1117 cm^−1^. According to literature, these peaks can be assigned to asymmetric P−O stretching vibrations of PO_4_^3−^ ions, thus confirming the presence of the phosphate coating.^[42]^ In principle, for a free phosphate ion, which belongs to the T_d_ point group, only one signal for the triply degenerate asymmetric P−O stretching vibration in the range 1100–1000 cm^−1^ would be expected. However, the interaction of PO_4_^3−^ with lithium cations or other ions can lower the symmetry and thus lead to peak splitting.^[43]^ The full spectra are shown in Figure S2, but no additional signals are observable.

**Figure 2:**
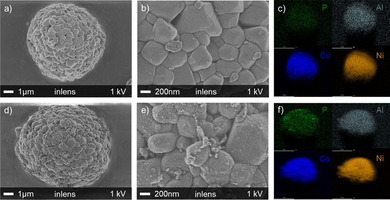
SEM images and EDS elemental mapping of pristine NCA (a–c) and c04‐NCA (d–f).

The morphology of pristine and coated NCA was investigated via scanning electron microscopy (SEM). Figure 2 displays the SEM images of pristine NCA (a–c) and c04‐NCA (d–f). Both samples (a,d) have spherical secondary particles, which are between 5–10 μm in diameter, made up of small primary particles that are 100–500 nm in diameter. The magnified images (b,e) show a very smooth surface for pristine NCA and a surface with an increased roughness for the coated material c04‐NCA. The energy‐dispersive X‐ray spectroscopy (EDS) mapping images show the elemental distribution on the particle surface (c,f). All the elements (Ni, Co, Al) of NCA show a homogenous distribution. Furthermore, phosphorus is also evenly distributed on c04‐NCA, which also supports the conclusion of a successful phosphate coating.

It is well‐known that the slurry pH increases into the highly alkaline region during a water‐based cathode electrode manufacturing process, which can lead to corrosion of the aluminum substrate. Therefore, the pH values of the pristine and coated NCA particles in aqueous mixtures were measured over a period of 2 h (Figure S3). Note that the pH results of pristine NCA were published previously.^[18]^ The pH of all samples rises almost instantaneously into the high alkaline region and then stabilizes after around 30 min. The pH value for pristine NCA is 12.69 after 2 h. This end pH value decreases with increased coating amount to 12.55, 12.43, 12.36, and 11.97 for c01‐NCA, c02‐NCA, c03‐NCA, and c04‐NCA, respectively. Since the pH value has a logarithmic dependency on the H_3_O^+^‐ion concentration, the differences in the pH measured result in a reduction of the OH^−^‐ion concentration by a factor of more than 7 for c04‐NCA. However, since all the pH values are higher than 9 and therefore beyond the regime where aluminum is passivated, the formation of basic species is only reduced, and aluminum corrosion and the concomitant generation of hydrogen gas can be expected during the electrode processing according to Equations (2) and 3.[^6^, ^44^]

(2) $$\mathrm{Al}{}_{2}O{}_{3}+2\;\mathrm{OH}{}^{-}+3\;H{}_{2}O\to 2\;\left[\mathrm{Al}\left(\mathrm{OH}\right){}_{4}\right]{}^{-}$$

(3) $$2\;\mathrm{Al}+2\;\mathrm{OH}{}^{-}+6\;H{}_{2}O\to 2\;\left[\mathrm{Al}\left(\mathrm{OH}\right){}_{4}\right]{}^{-}+3\;H{}_{2}$$

**Figure 3:**
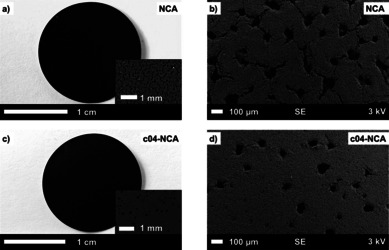
Photographs and SEM images of calandared aqueous‐processed electrodes with pristine NCA (a,b) and c04‐NCA (c,d).

Figure 3 displays photographs and SEM images of the top view of calandered aqueous‐processed electrodes with pristine and coated NCA (c04‐NCA). The data for the other electrodes (c01‐NCA, c02‐NCA, and c03‐NCA) can be found in Figure S4. As expected from the pH measurements, all electrodes show large holes and cracks stemming from the formation of hydrogen gas bubbles during aluminum corrosion.^[6]^ However, for the electrodes containing coated NCA, fewer holes are present (larger reduction for the electrodes with NCA with a higher coating amount), which suggests that the Al corrosion was at least partially suppressed. Moreover, this improvement can be also clearly seen with water‐based electrodes with a higher mass loading, where electrode cracking caused by hydrogen evolution is known to be a major issue (Figure S5).^[45]^ To fully compensate for the aluminum corrosion, a number of strategies present in the literature such as the addition of acids[^14^, ^46^] or amphoteric oxidic additives,^[47]^ CO_2_ gas treatment,[^8^, ^9^] and the use of carbon‐coated aluminum foil^[13]^ can be applied for further optimization. However, it should be noted that these approaches mitigate the consequences of water contact with the cathode material but do not prevent the origin of the problem from the beginning as is at least partially possible with a surface coating.

**Figure 4:**
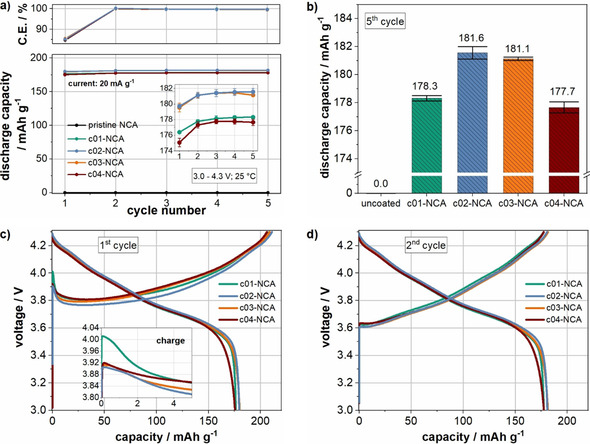
Results of the formation cycles of cells with aqueous‐processed electrodes: discharge capacity over cycle number (a), discharge capacity in the last formation cycle (b), and voltage profiles of a representative cell of each combination in the first and second cycle (c,d). The data in (a) and (b) represent the average specific discharge capacity of three cells, and the error bars relate to the standard deviation between these cells.

The electrochemical performance of cells containing the Li_3_PO_4_‐coated NCA in aqueous‐processed electrodes was investigated in pouch cells in a half‐cell configuration. The results of the formation are depicted in Figure 4. Figure 4a,b shows the average discharge capacity, while Figure 4c,d shows the voltage profiles of the first and second cycle, respectively. In accordance with a previous report of the authors, the cells with pristine NCA particles have a negligible electrochemical performance due to a high overpotential that exceeds the cut‐off voltage.^[18]^ The corresponding voltage profiles illustrate the extreme polarization of the cells (Figure S6). These results were attributed to the formation of water‐induced species, which cover the particle surface and severely hinder the extraction and insertion of lithium ions.^[15]^ In contrast, all cells containing coated‐NCA deliver a reasonable capacity, demonstrating a protective function of the Li_3_PO_4_ coating during the aqueous electrode processing. The initial specific discharge capacities are in the range of approximately 174–182 mAh g^−1^ at rate of C/10 with a coulombic efficiency of approximately 85 % (Figure 4a). As a comparison, the average specific discharge capacity value with NMP‐processed electrodes containing pristine NCA is approximately 192 mAh g^−1^. This result will be discussed in more detail later. Upon further cycling all cells show increasing specific discharge capacities with coulombic efficiencies above 99 %. The increasing capacity can be explained at least partially by a reversible Li^+^/H^+^‐exchange as suggested by Shkrob et al.^[48]^ This mechanism implies the formation of species such as NiOOH, which have been shown to be formed on the surface of water‐exposed NCA in a previous report.^[15]^ Moreover, mechanical stress induced by the volume expansion/contraction of NCA particles during lithium insertion and deinsertion might also contribute to the increase of capacity by enabling the electrolyte to wet previously not accessible pores.^[18]^ The specific discharge capacity of the last formation cycle (5th cycle) is a maximum of 181.6 mAh g^−1^ for c02‐NCA (Figure 4b). The capacity differs by less than 4 mAh g^−1^ between the four coated materials.

**Figure 5:**
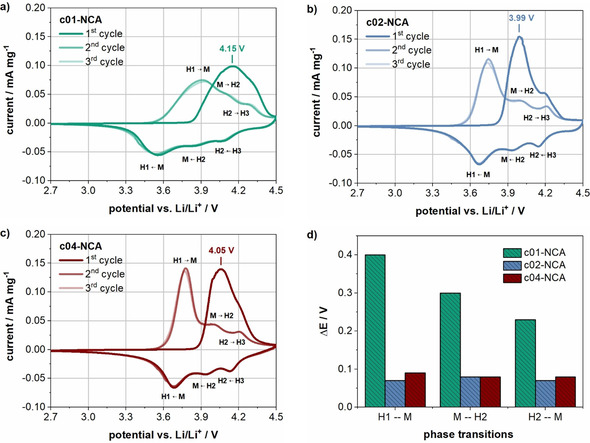
Cyclic voltammetry measurements at a scan rate of 0.05 mVs^−1^ between 2.7 and 4.5 V for three cycles of cells containing c01‐NCA (a), c02‐NCA (b), and c04‐NCA (c), and potential differences (Δ*E*) of NCA redox pairs in the third cycle (d).

The voltage profiles provide further information about the nature of the coating. The first and second cycle of a representative cell of each coated material is depicted in Figure 4c,d. All cells show an initial overvoltage as soon as the charge current is applied, which is the most pronounced for the cell with c01‐NCA followed by c04‐NCA, c03‐NCA, and c02‐NCA. The magnitude of the initial overvoltage has been reported to reflect the amount of water‐induced surface species on NCA particles.^[15]^ Moreover, various authors report a high initial overvoltage in cells containing nickel‐rich active materials or electrodes that have been exposed to moisture, which was ascribed to degradation of the surface or deposition of surface species.[^18^, ^39^, ^48^, ^49^] This can explain the high initial overvoltage for the lowest coating amount (c01‐NCA), where the particles seem to be the least protected against the exposure to water. In contrast, the cells containing a slightly higher amount of Li_3_PO_4_ in the coating (c02‐NCA) deliver the highest discharge capacity and lowest initial overvoltage. Finally, a coating amount that is “too high” results again in a higher initial overvoltage and lower discharge capacity as seen for c03‐NCA and c04‐NCA. In accordance with the literature, the overvoltage at the beginning of the charge cycle is no longer present in the second cycle, which was explained by at least partial decomposition of the surface species during the first cycle.[^15^, ^18^, ^39^, ^50^] In an effort to better understand the different behavior of the cells during the formation, cells with c01‐NCA, c02‐NCA, and c04‐NCA, as examples for a low, medium, and high Li_3_PO_4_ coating amount, were selected for cyclic voltammetry and impedance spectroscopy measurements. Figure 5a–c shows the cyclic voltammetry curves of the c01‐NCA, c02‐NCA, and c04‐NCA electrodes in lithium half‐cells at a scan rate of 0.05 mV s^−1^ between 2.7 and 4.5 V. The first cycle clearly differs from cycles two and three. In the first cycle, there is a main oxidation peak at 4.15, 3.99, and 4.05 V for the cells containing c01‐NCA, c02‐NCA, and c04‐NCA, respectively. Thus, the voltage of this first peak is highest for electrodes with the lowest amount of coating (c01‐NCA) followed by the highest (c04‐NCA) and medium (c02‐NCA) Li_3_PO_4_ coating amount. This trend is consistent with the order of the initial overvoltage observed in the first cycle of the galvanostatic formation (Figure 4c). In the second and third cycles, the characteristic redox pairs of NCA appear, which are attributed to the phase transitions from hexagonal (H1) to monoclinic (M), monoclinic (M) to hexagonal (H2), and hexagonal (H2) to hexagonal (H3) during lithium extraction.^[51]^ Figure 5d shows the potential differences (Δ*E*) between the anodic and cathodic scans of the three redox pairs for each cell variant in the third cycle. While the cells with c02‐NCA and c04‐NCA show comparable Δ*E* values (lowest for c02‐NCA), they are considerably higher than those for c01‐NCA, indicating an increased cell polarization.^[52]^ Another sign for an increased cell polarization for the lowest coating amount (c01‐NCA) is that the anodic peaks for H1/M and M/H2 are not clearly separated, in contrast to the cells with c02‐NCA and c04‐NCA, but are partially overlapped.^[53]^

**Figure 6:**
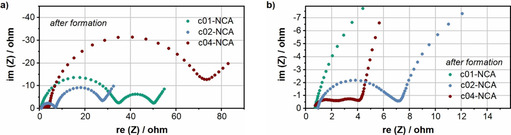
Electrochemical impedance spectra of cells containing c01‐NCA, c02‐NCA, and c04‐NCA at 4.3 V after the formation protocol: full spectra (a) and magnified view of the high‐frequency regime (b).

Electrochemical impedance spectroscopy was conducted after the formation cycles to gain more understanding of the effect of the amount of coating. The results obtained for c01‐NCA, c02‐NCA, and c04‐NCA are depicted in Figure 6. For c01‐NCA and c02‐NCA, the Nyquist plots consist of four features, which are assigned based on studies of similar cathode materials in the literature as the following: a high‐frequency intercept due to ohmic resistances within the cell (*R*_s_), a first semicircle related to surface film impedance (*R*_f_), a second semicircle related to charge‐transfer impedance at medium‐low frequencies (*R*_ct_), and an inclined line ascribed to solid‐state diffusion at low frequencies.^[54]^ In addition, for c04‐NCA a third semicircle appears in the mid‐frequency region, which can be correlated based on previous literature to the charge‐transfer resistance at the metallic lithium/electrolyte interface along with the electronic conductivity of the active material (R_*_).^[55]^ Therefore, a fit model with an additional element was used for c04‐NCA (Figure S7, Table S1). For c01‐NCA and c02‐NCA, it is possible that contributions associated with *R*_f_ and R_*_ are superimposed in the first semicircle. However, as these contributions cannot be separated by the eye, they were fitted with a single element.

As expected, the ohmic resistance (*R*_s_) is similar for all three cell types. However, the surface film impedance is much higher for the low coating variant (c01‐NCA) as compared to the medium coating amount (c02‐NCA) and is lowest for the high coating amount (c04‐NCA). This result may suggest that as the amount of coating increases, the active material particles are better protected during aqueous electrode processing, and that negative effects of water contact such as delithiation, degradation of the active material structure, as well as the deposition of surface species are partially suppressed. In contrast, *R*_ct_ is lowest for c01‐NCA, slightly higher for c02‐NCA, and much higher for c04‐NCA. According to literature, a thick or overly‐dense coating of a material with poor electronic conductivity can increase the charge‐transfer resistance.[^30^, ^54^] Keeping in mind that lithium phosphate can also be used as a solid electrolyte^[37]^ because of its low electronic conductivity, this implies that for a Li_3_PO_4_ coating a compromise between a reduction of the surface film resistance *R*_f_ and an increased charge transfer resistance *R*_ct_ has to be made, which seems to be the case with the medium coating amount (c02‐NCA).

**Figure 7:**
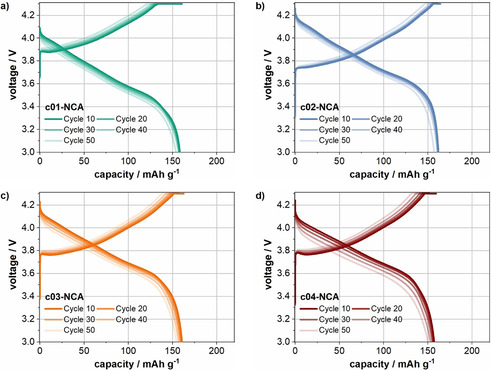
Voltage profiles of a representative cell of c01‐NCA (a), c02‐NCA (b), c03‐NCA (c), and c04‐NCA (d) during cycling.

The cycling results of the various cells at 25 °C with an initial cycle at C/10 followed by 49 cycles at a rate of 1 C in the voltage range of 3.0–4.3 V are shown in Figure 7 and Figure S8. Not surprisingly, the cells containing the pristine NCA still do not deliver any capacity (Figure S8). At first glance, the electrochemical performance of the cells with coated NCA differ only marginally, and all show relatively stable cycling. The discharge capacities at 1 C are in the range of 145–165 mAh g^−1^ during the whole cycling procedure.

The cells with c02‐NCA have a slightly higher discharge capacity than the other three coated NCA materials. The corresponding voltage profiles can be found in Figure 7a–d. The cell polarization is lowest for the cells with the coated materials c02‐NCA followed by c03‐NCA, c04‐NCA, and c01‐NCA, and increases with increasing cycle number. Moreover, the length of the CV‐step, and thus the capacity delivered during the CV step, is the highest for the cells with c01‐NCA. Specifically, the constant‐voltage phase delivers approximately 17 % and 24 % of the capacity in the first and last 1 C cycle, respectively. In comparison, the capacity delivered in the CV step for the materials with a higher coating content is 4 %/8 %, 6 %/13 %, and 7 %/15 % for the first/last 1 C cycles of c02‐NCA, c03‐NCA, and c04‐NCA, respectively. These results are consistent with those of Jung et al., who reported that the capacity obtained in the CV step was significantly higher in cells with NMC811 aged under ambient conditions as compared to cells with fresh NMC811.^[39]^

**Figure 8:**
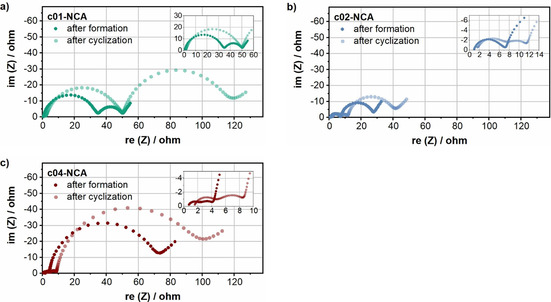
Comparison of electrochemical impedance spectra (a) of cells containing c01‐NCA, c02‐NCA, and c04‐NCA at 4.3 V after formation and after cycling. The high‐frequency region is magnified in the inset for each material.

The impedance was also compared for the same materials as above after cycling (Figure 8). Unlike the results after the formation cycles, a clear semicircle for R_*_ is now also visible for c02‐NCA. *R*_f_ after cycling increased by approximately the same factor for all cells, and the trend is the same as after formation (c04‐NCA<c02‐NCA<c01‐NCA; Table S2). In contrast, *R*_ct_ significantly increases for c01‐NCA over cycling by a factor of approx. 4, but it only slightly increases for c02‐NCA and c04‐NCA. According to the literature, a smaller increase for *R*_ct_ is consistent with reduced side reactions during cycling.^[56]^ The origin for reduced side reactions may be explained by the following considerations. The coated materials c02‐NCA and c04‐NCA seem to be better protected against water exposure and therefore the deposition of surface species. These surface species may react with ethylene carbonate (EC)‐containing electrolytes during cycling and lead to increased resistances.^[40]^ Furthermore, an optimized coating layer might act as a barrier between NCA and the electrolyte, which may hinder electrolyte decomposition. This aging mechanism is known to be one of the major issues for Ni‐rich layered cathode materials that leads to increased cell degradation.[^57^, ^58^] This benefit has been already reported by different authors for a Li_3_PO_4_ coating used in combination with NMP‐based electrode processing.[^24^, ^59^, ^60^] It should be noted that *R*_ct_ remains the highest for c04‐NCA even after cycling, which, as mentioned before, is mainly attributed to the low electronic conductivity of the coating. It seems that to achieve the best electrochemical performance, c02‐NCA is the coating amount of choice, at least from those tested, as reflected by the lowest overall impedance both after formation and after cycling.

**Figure 9:**
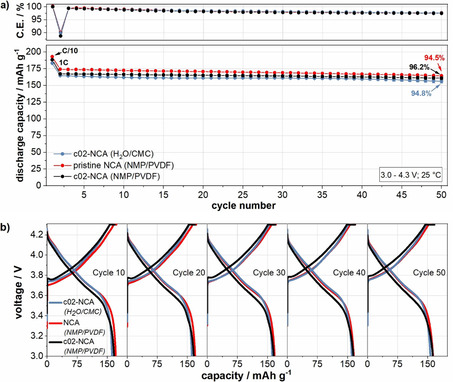
Cycle performance of aqueous‐ and NMP‐processed cathodes (a) and corresponding voltage profiles of a representative cell of each combination (b). The aqueous‐processed cathodes contain c02‐NCA, while the conventional cathodes contain pristine NCA and c02‐NCA, respectively. The data in (a) represent the average specific discharge capacity of three cells, and the error bars relate to the standard deviation between these cells.

Finally, the cell performance of the optimized water‐based electrodes with c02‐NCA was compared to that of cells containing conventional NMP/polyvinylidene fluoride (PVDF)‐based electrodes with pristine NCA and c02‐NCA. The data of the cells with NMP‐electrodes with pristine NCA was previously published by these authors.^[15]^ The formation results for the cells with the conventional electrodes are shown in Figures S9 and S10. During cycling (Figure 9a,b), the cells with NMP‐processed electrodes containing pristine NCA deliver an average specific discharge capacity of 192.8 mAh g^−1^ in the initial C/10 cycle and 174.0 mAh g^−1^ in the first 1 C cycle. These values are slightly higher than for the cells with NMP‐based electrodes containing c02‐NCA (188.2 mAh g^−1^ at C/10 and 167.2 mAh g^−1^ at 1 C). In addition, the associated voltage profiles show an increased cell polarization for the NMP‐based c02‐NCA variant (Figure 9b). This might be explained by the presence of the coating that was optimized for an aqueous electrode manufacturing process rather than for an NMP/PVDF‐based one. The latter does not require the protection of particles against water exposure and will likely also result in different cathode particle/binder interactions [PVDF vs. sodium carboxymethyl cellulose (CMC)].[^1^, ^61^] Therefore, higher capacity values and lower cell polarization might be obtained by optimizing the coating amount for an NMP/PVDF based process, which is beyond the scope of this work. Compared to the NMP/PVDF variants, the capacity values of the cells with aqueous‐processed c02‐NCA electrodes are between 5–10 mAh g^−1^ lower (183.0 mAh g^−1^ at C/10 and 164.8 mAh g^−1^ at 1 C). However, the capacity retention of these cells at 1 C is 94.8 % and therefore slightly higher than the NMP‐based variant with pristine NCA (94.5 %). The cells with NMP‐processed electrodes containing c02‐NCA have an even higher capacity retention of 96.2 %. These trends suggest that the Li_3_PO_4_ coating may provide additional protection against side reactions during cycling as it was reported in previous literature.[^24^, ^59^, ^60^] Another plausible interpretation for these differences is that for the cells with higher capacity values, where more lithium ions were extracted from the NCA structure (i. e., a higher state of charge), this can lead to enhanced degradation of the NCA particles and result in a more rapid deterioration of the cell performance.[^57^, ^62^] Although there are different mechanisms to explain the differences in the capacity retention, the aqueous processing certainly plays a role in slightly lower retention of the cells with the aqueous‐processed electrodes as compared to these with NMP‐processed coated NCA. The coating amount with c02‐NCA is a compromise, as described above. Therefore, the formation of a small amount of water‐induced surface species likely occurs even here, which may also contribute to additional side reactions.^[15]^ Furthermore, an impact of the different binder properties of PVDF and CMC should be considered.[^1^, ^61^] More studies are needed to clarify this point in detail.

Currently, the electrochemical performance of full cells containing optimized water‐based NCA electrodes is under investigation to demonstrate long‐term cycling stability, and an upscaling process for the coating procedure is developed.

## Conclusions

By a simple precipitation reaction, LiNiCoAlO_2_ (NCA) particles were coated with different amounts of lithium phosphate. The use of the coated NCA in aqueous electrode processing reduced the alkalinity of the slurry and thus the lithium leaching in water could be at least partially suppressed. In electrochemical testing, all cells with coated NCA showed good electrochemical performance. The results suggest that the coating amount is crucial to optimize the cell performance. A low coating amount leads to relatively poor protection of the NCA during aqueous processing. As a result, more pronounced structural damage and/or deposition of surface impurities occur, which is reflected by a high initial overvoltage in the first charge and an increased surface film impedance. In contrast, a lithium phosphate coating amount that is too high provides better protection against water but leads to increased electronic resistance. Therefore, a compromise has to be made between protection against water and sufficient electronic conductivity through the particle surface.

The best‐performing cells containing aqueous‐processed electrodes were compared with cells with *N*‐methyl‐2‐pyrrolidone (NMP)‐based electrodes. Although the NMP cells have slightly higher discharge capacities, the water‐based variant can compete in terms of cycle life. Although further studies are needed, this work demonstrates that applying protective surface coatings on NCA particles can be a feasible way towards a successful implementation of an aqueous‐based electrode processing for this water‐sensitive material.

## Experimental Section

### Li_3_PO_4_ coating

Commercial LiNi_0.8_Co_0.15_Al_0.05_O_2_ (TODA, NAT‐1050) was used as received. For the Li_3_PO_4_ coating, the appropriate amount of lithium acetate dihydrate (Sigma Aldrich, 98 %) was dissolved in ethanol (20 g). NCA (10 g) was added and the suspension was homogenized for 10 min at room temperature. Dropwise, a solution consisting of phosphoric acid (Merck, 85 wt % in water) in 5 g ethanol was added within 15 min under vigorous stirring. The molar ratio between lithium acetate dihydrate and phosphoric acid was controlled to be 3 : 1. Next, the suspension was dried under reduced pressure, and the powder was annealed at 300 °C for 5 h in ambient atmosphere (heating rate: 5 °C min^−1^) to remove residual organic compounds. To evaluate the effect of the coating, four different samples with varying Li_3_PO_4_‐amounts per g NCA (0.00625 mmol g^−1^, 0.0125 mmol g^−1^, 0.025 mmol g^−1^, and 0.05 mmol g^−1^) were prepared assuming that all precursors were totally converted to Li_3_PO_4_ after the coating process. The surface modified NCA‐particles are referred to by increasing coating amount as c01‐NCA, c02‐NCA, c03‐NCA, and c04‐NCA, respectively.

### Electrode processing

CMC (Sigma‐Aldrich, mass average molar mass ≈250000 g mol^−1^ with a degree of substitution of 0.9) and Super C65 (Imerys) were used as binder and conductive carbon, respectively. Slurries consisting of 92 wt % active material, 4 wt % CMC, and 4 wt % Super C65 in deionized water were prepared by the following steps: CMC was dissolved in deionized water using a laboratory shaker. After the addition of either the pristine or the coated NCA and Super C65, the mixture was homogenized by a speedmixer. Using the doctor‐blade technique, the slurries were coated onto aluminum foil and then pre‐dried at 80 °C for 30 min. The resulting electrode sheets were calandered (50 % of initial electrode thickness), and disc electrodes with an area of 2.01 cm^2^ were punched out (mass loading: 11±1 mg cm^−2^). To remove residual moisture, the disc electrodes were dried under vacuum at 110 °C for 10 h.

To prepare NMP‐based electrodes, PVDF (Solvay, Solef® 5130) was dissolved in NMP (Sigma‐Aldrich) overnight. Then, the active material and conductive carbon (Imerys, Super C65) were added, and the mixture was homogenized in a speedmixer. The ratio of active material/conductive carbon/binder was kept the same as for the aqueous route. After casting the slurries on aluminum foil, the electrodes were stored in a fume hood overnight and pre‐dried under vacuum (80 °C, 2 h). The dried electrode sheets were calendered to 50 % of initial electrode thickness. Disc electrodes (16 mm in diameter) with a mass loading of 11±1 mg cm^−2^ were punched out and dried under vacuum at 80 °C for 5 h.

### Cell assembly and electrochemical measurements

Pouch cells with the NCA electrodes, lithium metal (Sigma‐Aldrich) on a copper substrate as counter electrode, a polyethylene separator (Celgard 2500) and 1 mol L^−1^ LiPF_6_ in EC/DMC (dimethyl carbonate) 1 : 1 *w*/*w* (LP30, BASF) as electrolyte were assembled in an argon filled glovebox (MBraun). To contact the electrodes, aluminum tabs (Targray) and copper‐nickel tabs (Targray) were used for the cathode and anode, respectively. To ensure data reliability three cells were prepared for each variant.

Charge/discharge tests were conducted on an electrochemical workstation (Maccor, Series 4000) in the voltage range of 3.0 to 4.3 V at 25 °C (climate chamber, Memmert). The cells were activated with a formation protocol consisting of five cycles at C/10 (20 mA g^−1^), whereby charge and discharge were done in constant‐current mode (CC). The capacity of the 5th formation cycle was used to calculate the current for the cycling test, which consisted of one cycle at C/10 and 49 cycles at 1 C. Here, the charge was done with a constant‐current constant‐voltage (CCCV) process and discharge was carried out in the CC‐mode. The CV‐step was terminated at a current of C/20. Due to the low coating amount, the coated NCA particles were treated as pure active material for the specific capacity calculations. This may artificially reduce the actual specific capacity of the active material. Cyclic voltammetry tests and electrochemical impedance spectroscopy (EIS) were conducted on a VMP300 galvanostat/potentiostat (BioLogic). Impedance spectra were obtained by the perturbation of the cells with an AC voltage (amplitude: 5 mV) over the frequency range of 1 MHz to 10 mHz. Cyclic voltammograms were recorded between 2.7 and 4.5 V with a scan rate of 0.05 mV s^−1^ at 25 °C (climate chamber, Memmert). For these measurements a lithium reference electrode was added to the cell setup described above.

### Characterization methods

The crystal structures of the active material powders were analyzed by XRD (CuK_α_: *λ*=0.1540598 nm; PANalytical Empyrean series 2) in the 2*θ*‐range 10–80° (step size: 0.003°, aperture: 10 mm). ATR‐FTIR analysis was performed using an Alpha II (Bruker) spectrometer with germanium crystal in the range of 700–4000 cm^−1^. All spectra were recorded with 64 scans and a resolution of 2 cm^−1^. The spectra were normalized to the largest peak. To measure the pH values a pH meter (pH 315i, WTW) equipped with a SenTix®H electrode (WTW) was used. The pristine or coated NCA was added to distilled water (mass ratio NCA/water=1 : 3) and the pH evolution with continuous stirring by a magnetic stirrer (750 rpm) was monitored for 2 h. The surface morphology and elemental distribution was investigated by SEM (ZEISS Ultra 55, Carl Zeiss Microscopy GmbH) coupled with EDS.

## Conflict of interest

The authors declare no conflict of interest.

## Supporting information

As a service to our authors and readers, this journal provides supporting information supplied by the authors. Such materials are peer reviewed and may be re‐organized for online delivery, but are not copy‐edited or typeset. Technical support issues arising from supporting information (other than missing files) should be addressed to the authors.

Supplementary
