# Supplementary material for: Surface Modification of LiNi0.8Co0.15Al0.05O2 Particles via Li3PO4 Coating to Enable Aqueous Electrode Processing
Source: ChemSusChem. 2020 Oct 7;13(22):5962–71. doi: 10.1002/cssc.202001907 (PMC7756629; doi:10.1002/cssc.202001907)
Supplement: Supplementary file 1 — Supplementary [file CSSC-13-5962-s001.pdf]

ChemSusChem

Supporting Information

Surface Modification of $LiNi_{0.8}Co_{0.15}Al_{0.05}O_2$ Particles via Li_3PO_4 Coating to Enable Aqueous Electrode Processing

Michael Hofmann, Felix Nagler, Martina Kapuschinski, Uwe Guntow, and Guinevere A. Giffin*© 2020 The Authors. ChemSusChem published by Wiley-VCH GmbH. This is an open access article under the terms of the Creative Commons Attribution License, which permits use, distribution and reproduction in any medium, provided the original work is properly cited.

Author Contributions

M.H. Conceptualization:Lead; Investigation:Lead; Methodology:Lead; Writing - Original Draft:Lead
F.N. Investigation:Supporting; Methodology:Supporting
M.K. Investigation:Supporting
U.G. Conceptualization:Supporting; Funding acquisition:Lead; Investigation:Supporting; Methodology:Supporting; Supervision:Supporting; Writing - Review & Editing:Supporting
G.G. Conceptualization:Supporting; Investigation:Supporting; Methodology:Supporting; Supervision:Lead; Writing - Review & Editing:Lead

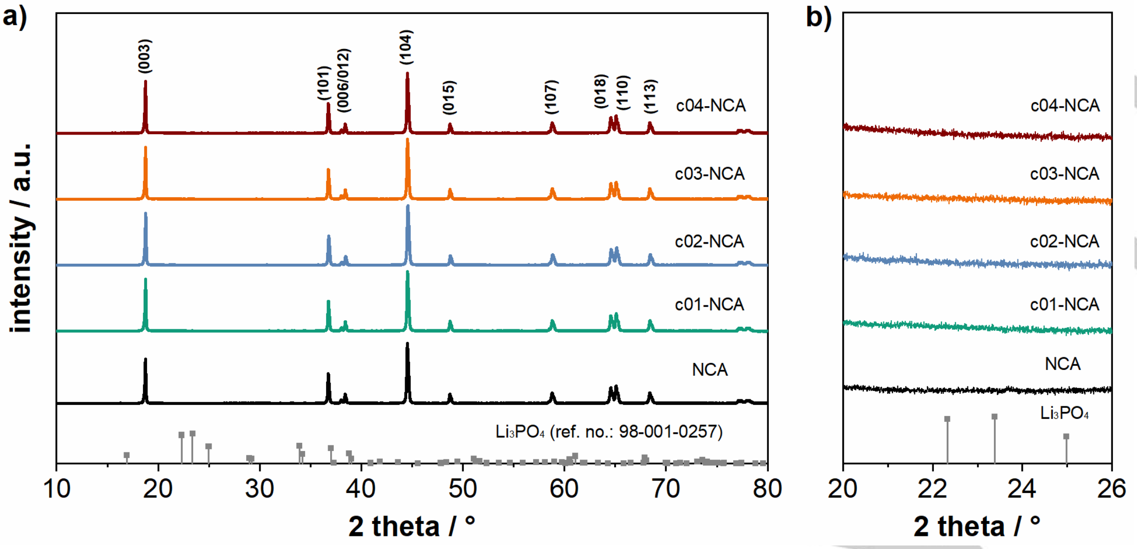

Figure S1 XRD patterns of pristine and coated NCA-particles (a), and a magnified 2θ range of 20 – 26 ° (b).

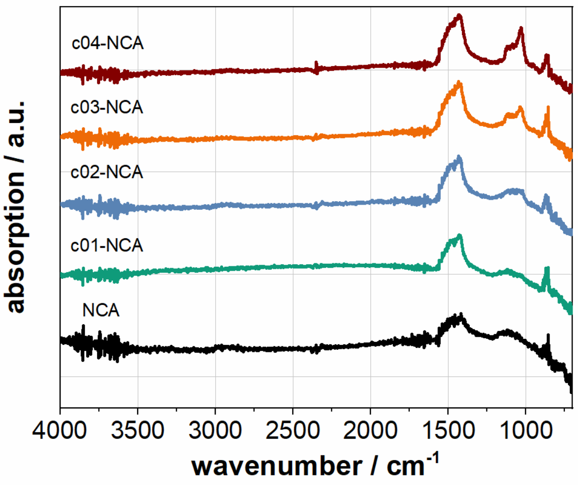

Figure S2 ATR-FT-IR spectra of pristine and coated NCA-particles.

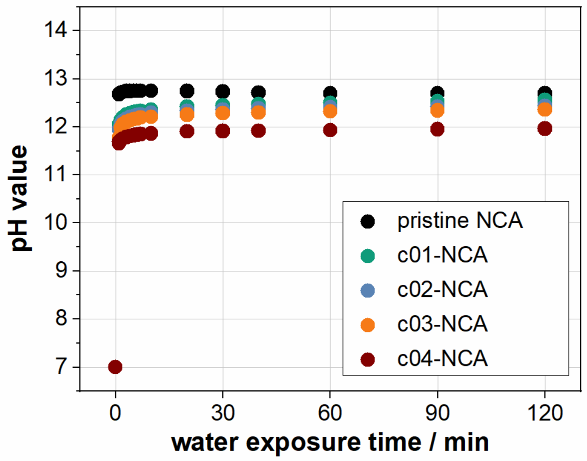

Figure S3 pH measurements of pristine and coated NCA-particles in water over a period of two hours.

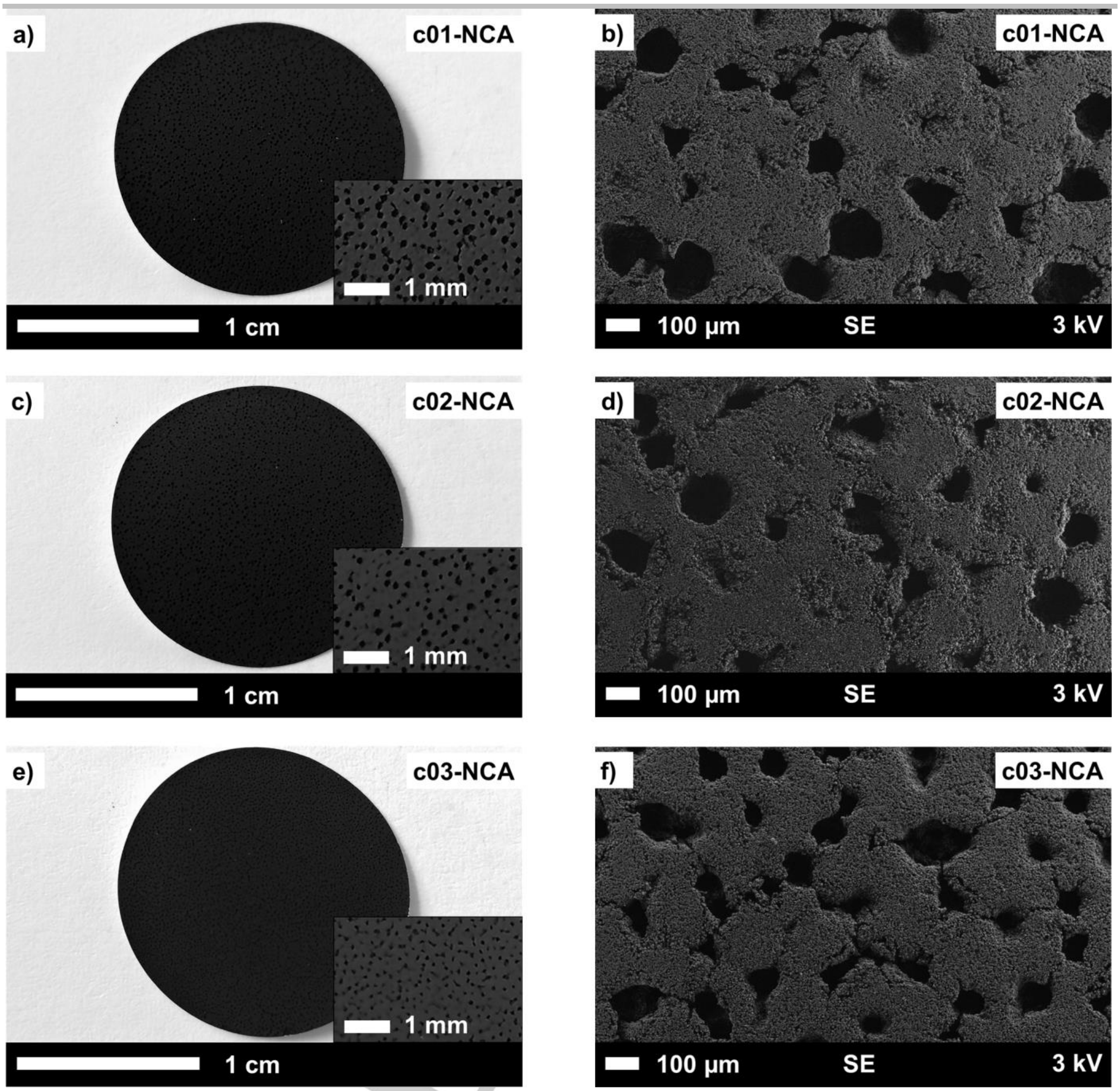

Figure S4 Photographs and SEM images of the top view of calandered aqueous-processed electrodes with c01-NCA (a, b), c02-NCA (c, d) and c03-NCA (e, f).

WILEY

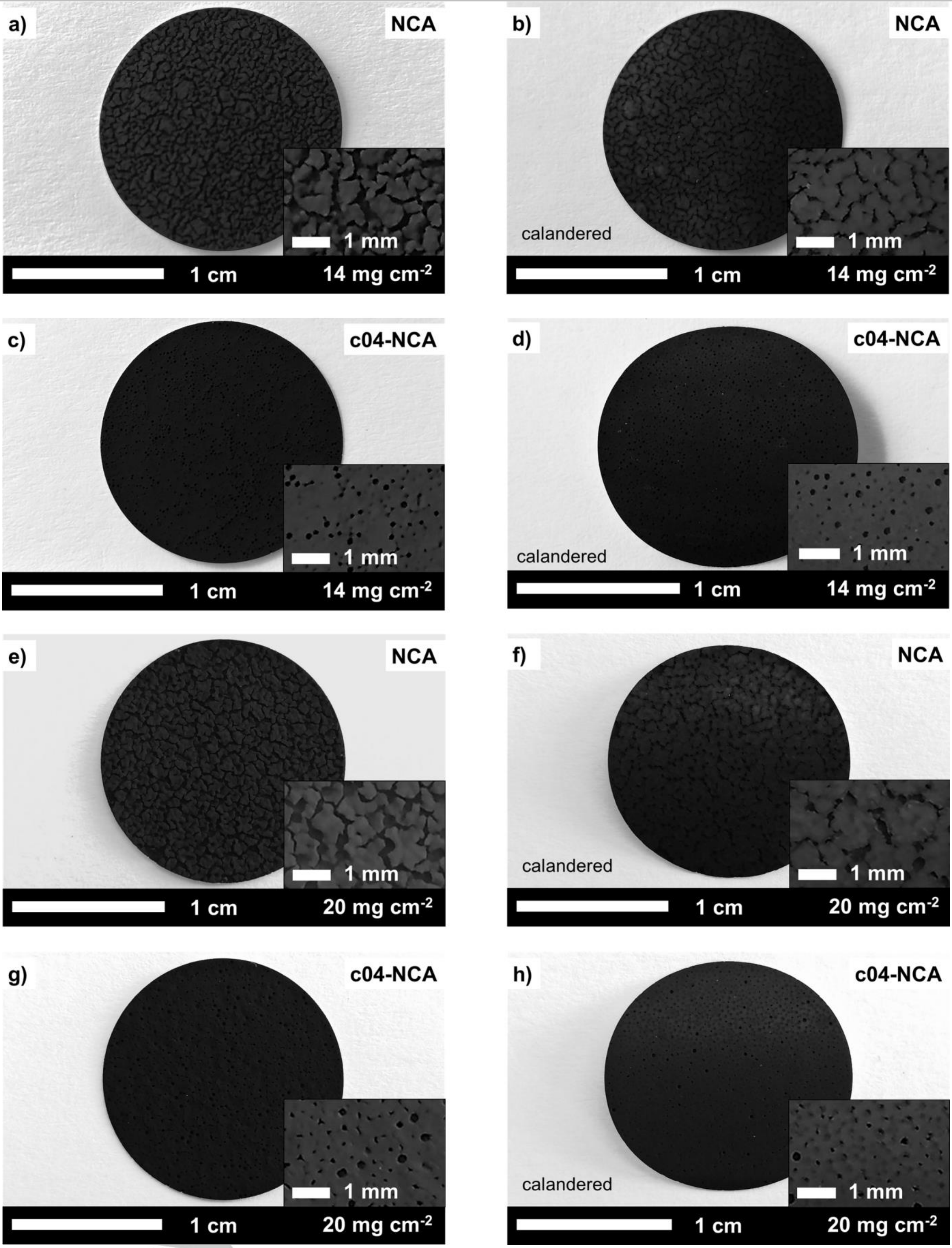

Figure S5 Photographs of uncalandered and calandared aqueous-processed electrodes with different mass loadings containing pristine NCA (a, b, e, f) and c04-NCA (c,d, g, h).

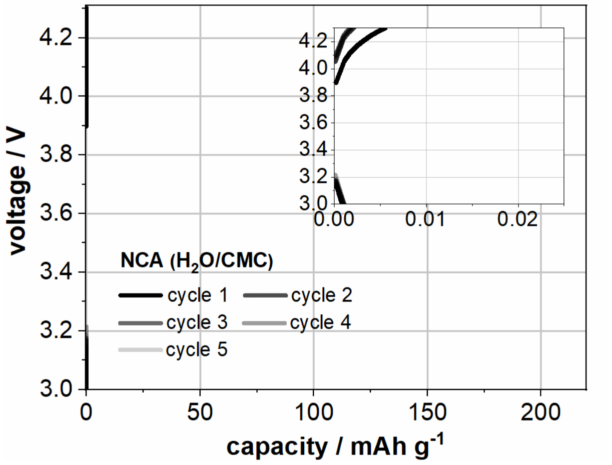

Figure S6 Voltage profiles during formation of a representative cell with aqueous-processed NCA electrodes.

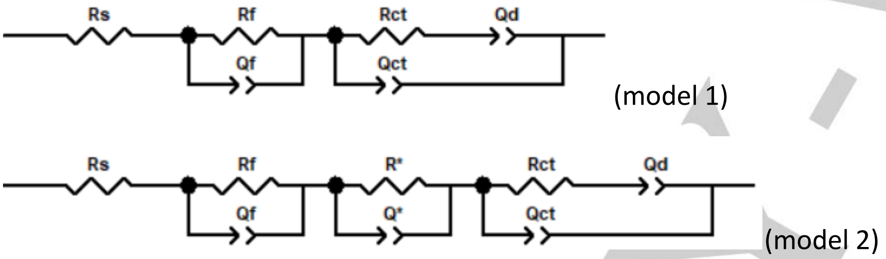

Figure S7 Equivalent circuit models used for fitting of EIS spectra. The various elements in model 1 are attributed to the following processes: ohmic resistances within the cell (resistor R_s), surface film impedance and capacity (RCPE-element Rf, Qf), charge transfer impedance and double layer capacity (RCPE-element Rct, Qct) and solid-state diffusion (CPE-element Qd). The additional RCPE-element (R*, Q*) in model 2 is used to describe the charge transfer process at the metallic lithium/electrolyte interface along with the electronic conductivity of the active material. The information which model was used for the fit can be found in Table S 1 and S 2.

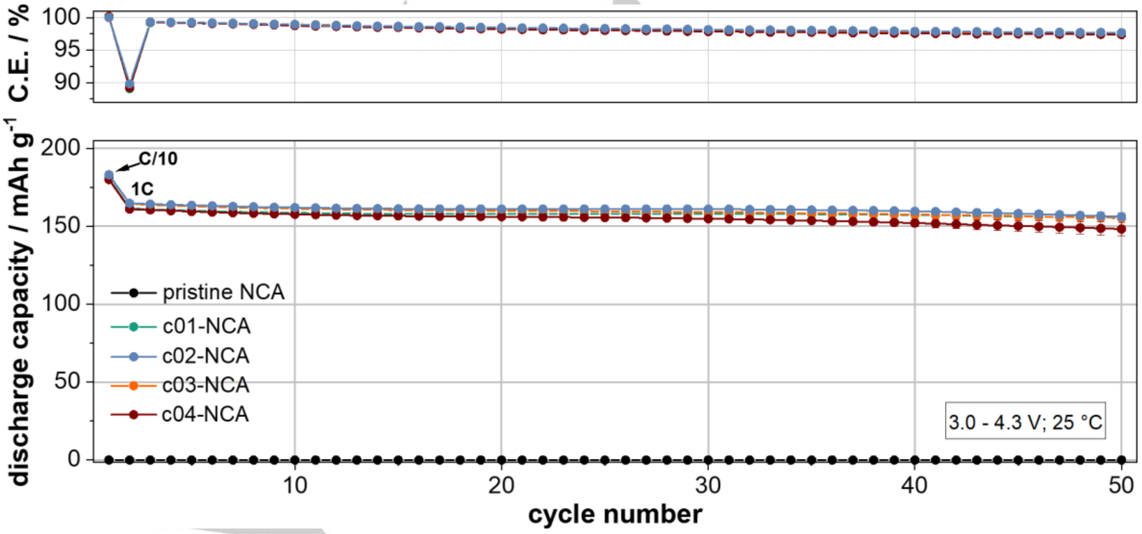

Figure S8 Cycling of half-cells with aqueous-processed electrodes between 3.0 – 4.3 V. The data represent the average specific discharge capacity of three cells and the error bars relate to the standard deviation between these cells.

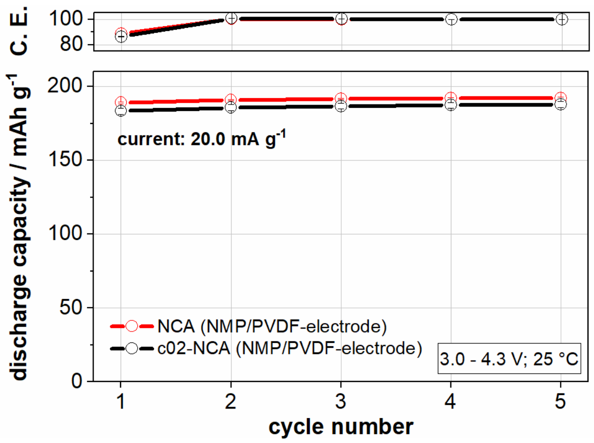

Figure S9 Discharge capacity during formation for cells with NMP-processed electrodes containing pristine NCA and c02-NCA, respectively.

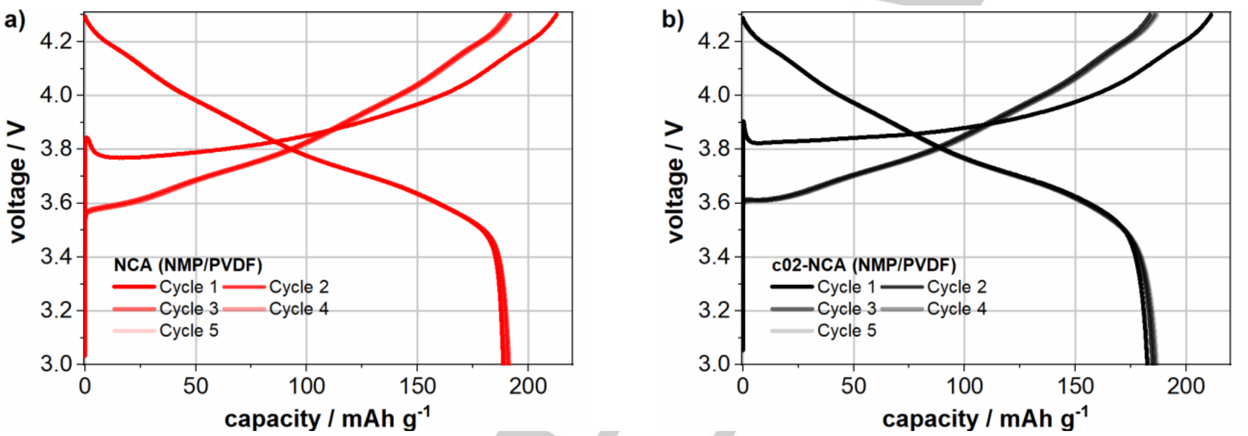

Figure S10 Voltage profiles during formation for a representative cell with NMP-processed electrodes containing pristine NCA (a) and c02-NCA (b).

WILEY-VCH

Table S1 Fitting results of EIS for cells containing c01-NCA, c02-NCA and c04-NCA after formation.

cell	R_s [Ω]	[%]	R_f [Ω]	[%]	R_* [Ω]	[%]	R_{ct} [Ω]	[%]	fit model
c01-NCA	0.76	± 0.80	33.11	± 0.21	-	-	16.94	± 1.27	model 1
c02-NCA	0.93	± 1.98	6.08	± 0.91	-	-	21.44	± 1.87	model 1
c04-NCA	0.64	± 2.85	1.54	± 3.26	2.29	± 1.98	67.27	± 0.35	model 2

Table S2 Fitting results of EIS for cells containing c01-NCA, c02-NCA and c04-NCA after cycling.

cell	R_s [Ω]	[%]	R_f [Ω]	[%]	R_* [Ω]	[%]	R_{ct} [Ω]	[%]	fit model
c01-NCA	2.01	± 0.88	48.05	± 0.33	-	-	66.92	± 2.52	model 1
c02-NCA	1.53	± 0.38	5.85	± 0.54	4.44	± 0.92	28.00	± 0.33	model 2
c04-NCA	1.52	± 0.69	3.24	± 0.95	4.14	± 0.84	86.82	± 0.47	model 2

WILEY-VCH
